# Association between serum vitamin D level and Graves’ disease: a systematic review and meta-analysis

**DOI:** 10.1186/s12937-024-00960-2

**Published:** 2024-06-07

**Authors:** Boxian Pang, Leyang Li, Xin Liu, Zhengmin Cao, Tieliang Pang, Qiuhong Wang, Junping Wei

**Affiliations:** 1 Department of Endocrinology, Guang’anmen Hospital, China Academy of Chinese Medical Sciences, Beijing, China; 2 Graduate school, Beijing University of Chinese Medicine, Beijing, China; 3 Beijing Anding hospital, Capital Medical University, Beijing, China; 4 Infections Disease Section, Guang’anmen Hospital, China Academy of Chinese Medicine Sciences, Beijing, China; 5 Bioscience Department, University of Nottingham, Nottingham, UK

**Keywords:** Vitamin D, Graves’ disease, Systematic review, Meta-analysis

## Abstract

**Objective:**

This meta-analysis aims to analyze the relationship between serum vitamin D (VD) levels and Graves’ disease (GD).

**Methods:**

We conducted a search for publications on VD and GD in the English language. Our search encompassed databases such as PubMed, Embase, Web of Science, and the Cochrane Library, covering publications available through August 2023. A meta-analysis was performed using Cochrane RevMan 5.4 software. The standardized mean difference (SMD) and 95% confidence interval (CI) were used for outcome calculation. We used R software to test for publication bias.

**Results:**

Twelve studies were selected, comprising 937 (22.4%) cases with GD and 3254 (77.6%) controls. The overall meta-analysis revealed that patients with GD are significantly more likely to have low VD levels (SMD = − 0.66; 95% CI: −1.05, − 0.27; *p* = 0.001) than those in the control group. Egger’s test results indicated no publication bias (*p* = 0.0791). These studies exhibited a high degree of heterogeneity (chi-square = 205.86, *p* < 0.00001; I^2^ = 95%). Subgroup analysis was conducted based on assay method, geographic location, and mean age of the case group to explore the heterogeneity sources. Assay methods and geographic locations were identified as potential heterogeneity sources. Based on the mean age, there were no statistically significant differences found in the subgroup analysis of the included studies.

**Conclusion:**

There is promising evidence that low serum VD levels may increase the risk of GD. Further rigorous and long-term trials are needed to explore the role of VD in the onset and treatment of GD.

## Introduction

Graves’ disease (GD) is an organ-specific autoimmune disease characterized by elevated levels of thyrotropin receptor antibodies (TRAb). These antibodies activate the thyroid stimulating hormone (TSH) receptor, leading to increased production and release of thyroid hormones, ultimately resulting in hyperthyroidism [1]. GD is the primary cause of hyperthyroidism, affecting 20–50 cases per 100,000 people [2]. Common clinical manifestations include tremors, weight loss despite normal appetite, anxiety and irritability, enlargement of the thyroid gland (goiter), fatigue, frequent bowel movements, and palpitations. If left untreated, GD can result in atrial fibrillation, embolic events, neuropsychiatric symptoms, cardiovascular collapse, and death [3, 4]. Current treatment options for GD are limited to radioactive iodine (RAI) therapy, antithyroid drugs (ATDs), and thyroidectomy [5]. However, both RAI therapy and thyroidectomy can lead to hypothyroidism, necessitating lifelong thyroid hormone replacement [6], and ATDs carry a high risk of recurrence and cause adverse reactions such as liver function impairment and agranulocytosis [7]. Overall, GD is a harmful disease with a long treatment period, challenging to cure, and prone to recurrent attacks.

Vitamin D (VD), a seco-steroid hormone, was initially discovered during research on rickets prevention and treatment [8–10]. Nowadays, numerous studies have highlighted VD’s crucial roles in various immune system processes [11]. VD’s significance in the immune system stems from its close interaction with immune cells. It can modulate T cell function by inhibiting the release of cytokines such as interferon-γ (IFN-γ) and interleukin (IL)-17, while stimulating other cytokines, such as IL-4 [12]. Additionally, VD is closely associated with B cell function [13]. As a multifactorial autoimmune thyroid disease (AITD), GD is caused by the loss of central and peripheral immunological tolerance to thyroid antigens [11]. T and B lymphocytes play pivotal roles in AITD pathogenesis [14, 15], suggesting that VD influences the development of GD by regulating these immune cells.

Earlier studies have indicated that decreased VD levels can result in elevated thyroid stimulating antibody (TSAb) levels and thyroid gland enlargement [16, 17], suggesting a potential link between decreased VD levels and GD. However, some studies reported no correlation between serum VD levels and GD [18, 19], resulting in ongoing controversy regarding the impact of VD on the development of GD. Two meta-analyses published in 2015 examined the association between VD and GD, covering 13 and 26 studies, respectively [20, 21]. However, the inclusion of poor quality studies, including those no internationally peer-reviewed, such as those sourced from China National Knowledge Infrastructure (CNKI) dissertations, could have potentially introduced publication bias in these articles. Additionally, recent clinical trials exploring the impact of VD on the onset of GD have emerged in the past decade, providing updated research evidence. Therefore, our aim is to include high-quality, peer-reviewed studies to obtain more reliable evidence regarding the relationship between serum VD levels and the incidence of GD.

## Methods

### Search strategy

**Fig. 1:**
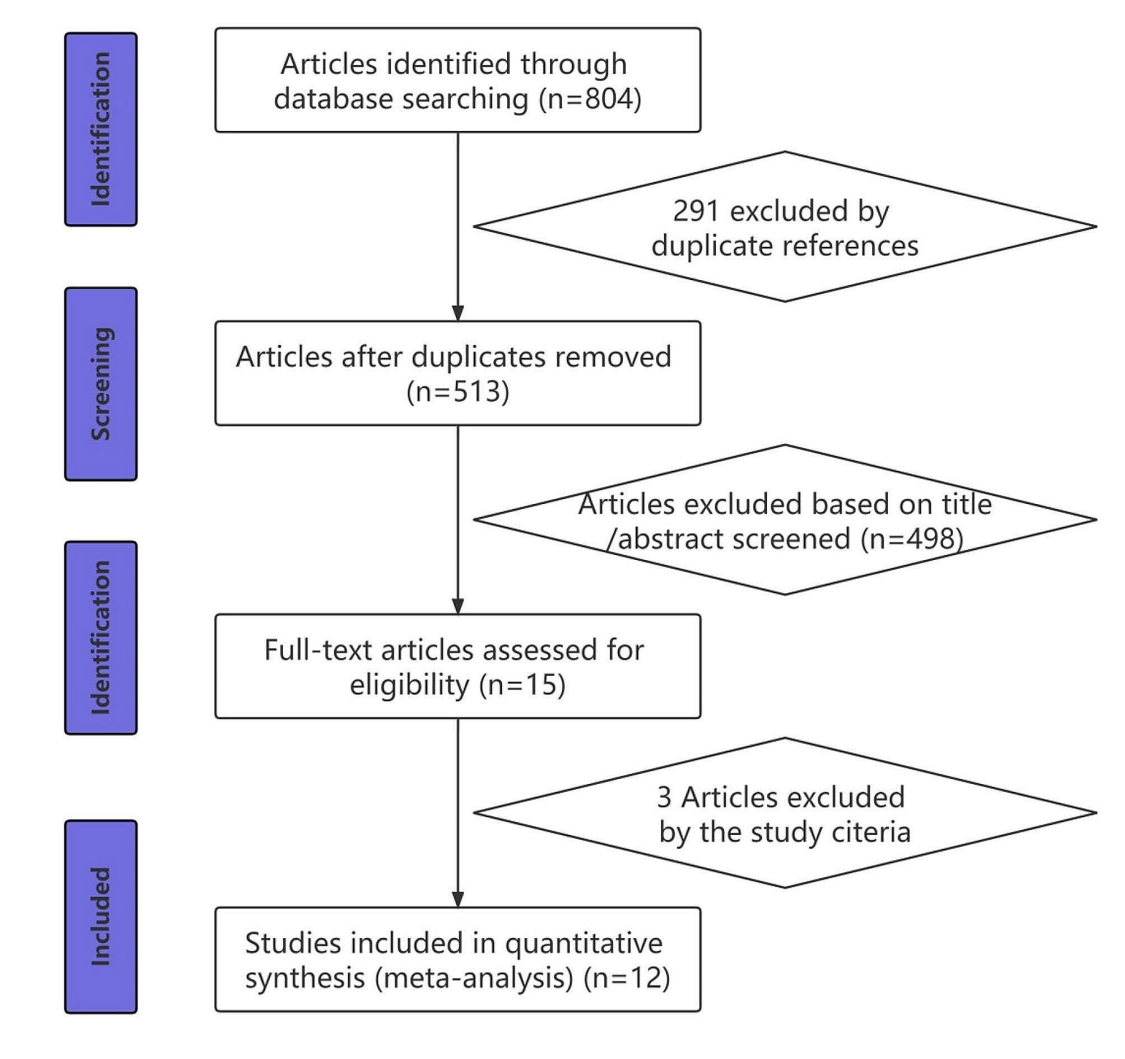
Flow chart of the literature selection

We conducted a literature search for relevant studies on VD and GD using PubMed, EMBASE, Web of Science, and the Cochrane Library (up to August 31, 2023). Two reviewers independently performed the searches using the following MeSH terms: “vitamin D,” “25 Hydroxyvitamin D,” “25 Hydroxyvitamin D3,” and “1,25 Hydroxyvitamin D3” in combination with “autoimmune thyroid disease” or “Graves’ disease.” The search strategy in the databases used the terms (“vitamin D” OR “25 Hydroxyvitamin D” OR “25 Hydroxyvitamin D3” OR “1,25 Hydroxyvitamin D3”) AND (“autoimmune thyroid disease” OR “Graves’ disease”). Only articles in English were included. Figure 1 illustrates the study selection process.

### Study selection

All studies were considered for review and meta-analysis if they met the following criteria: (1) Described a case–control study or cohort study; (2) included patients with GD in the case group and healthy individuals in the control group; and (3) provided quantitative data on VD. Studies were excluded from the meta-analysis if they met the following criteria: (1) not original research; (2) lack of clear VD data; (3) published as a dissertation or preprint; and (4) not meeting the high-quality study criterion.

### Data extraction and quality assessment

Information from the included studies was extracted using a standardized form by two reviewers. Any disagreements were resolved through discussion or consultation with a third investigator. The form included variables such as the first author’s last name, publication year, country, study year, mean age of case and control groups, detection index, assay method of serum VD levels, sample sizes of both groups, and serum VD levels (reported in ng/mL; nmol/L divided by 2.5 to convert to ng/mL). The quality of the studies was assessed using the Newcastle–Ottawa Quality Assessment Scale by two reviewers [22].

### Outcomes measures

The primary outcome was serum VD levels in the GD and healthy individuals groups, with a comparison of outcomes between the groups.

### Statistical analysis

Meta-analysis was conducted using Cochrane RevMan 5.4 software (Review Manager Version 5.4., The Cochrane Collaboration, 2020), and heterogeneity was assessed using I^2^ statistics. In terms of the I^2^ statistic, low, moderate, or high heterogeneity is represented by an I^2^ index of 25%, 50%, or 75%, respectively [23]. To reduce the impact of study’s heterogeneity, we opted for a random-effects model. Considering the same outcomes were assessed by different methods, this meta-analysis employed standardized mean difference (SMD) as a summary statistic. SMD and 95% confidence interval (CI) were calculated for outcome analysis. Subgroup analysis was performed when heterogeneity was present. Forest plots were used for data presentation, and statistical significance was set at *p* < 0.05. Funnel plots and Egger’s test in R software were used to assess the publication bias.

## Results

### Characteristics of included studies

Figure 1 presents a flowchart of the screening process. Initially, we identified *804* trials, of which *291* were duplicates. Following the screening of unduplicated papers based on abstract or title, 513 remained for full manuscript review, with 498 being disqualified based on their abstract or title. Finally, 15 reports underwent complete manuscript review, and 12 studies [24–35] were deemed eligible for inclusion. Table 1 presents the compiled features of these 12 studies.

Most studies were case–control studies, with sample sizes ranging from 84 to 2597, published in English between 1980 and 2020. All studies scored > 6, indicating high quality [22]. Scoring details are in the Supplementary material. Our meta-analysis included 4191 participants (937 [22.4%] GD cases and 3254 [77.6%] controls). Most studies were conducted in China [24, 28–31], with two in India [26, 33] and one each from the USA [25], Sweden [27], Japan [32], Germany [34], and Belgium [35].

Among the 12 studies, 10 recruited both males and females in the case and control groups, one included both males and females only in the case group [27], and one did not report sex [32]. All included studies assessed serum VD levels: eight assessed 25(OH)D, three assessed 25(OH)D_3_, and one did not specify the detection index and only mentioned VD [27]. Various methods were used to detect VD, including competitive protein binding assays (CPBA) [32, 33, 35], enzyme-linked immunosorbent assay (ELISA) [24, 31], electrochemiluminescence immunoassay (ECLIA) [26], radioimmunoassay (RIA) [29, 33], euglobulin clot lysis assay (ECLA) [28, 30], and high-pressure liquid chromatography (HPLC) [27]; one study did not report the method [25].

**Table 1:** Characteristics of the included studies

No.	First Author	Year	Country	Study Year	Patient Age (Mean ± SD)	Detection Index	Assay Method	Sample Size (Case/Control)	Quality Score
1	Feng [24]	2020	China	2017–2019	10.85 ± 2.79	25(OH)D	ELISA	54/30	7
2	Heisel [25]	2020	USA	2016–2018	50.1	25(OH)D	NA	89/356	8
3	Arun K [26]	2019	India	2015–2016	35.25 ± 9.70	25(OH)D	ECLIA	84/42	8
4	Tereza [27]	2018	Sweden	2017	NA	NA	HPLC	292/2305	7
5	Ke [28]	2017	China	2015–2016	39.79 ± 1.73	25(OH)D	ECLA	51/51	8
6	Li [29]	2015	China	2010	39.00 ± 8.06	25(OH)D	RIA (25(OH)D-Ria-CT)	32/60	9
7	Ma [30]	2015	China	2012–2013	40.04 ± 15.24	25(OH)D	ECLA	70/70	8
8	Zhang [31]	2015	China	2012	31.7 ± 10.32	25(OH)D	ELISA	70/70	9
9	Yasuda [32]	2013	Japan	2011	38 ± 7	25(OH)D_3_	CPBA	36/49	7
10	Jyotsna [33]	2012	India	2006–2008	36.33 ± 11.15	25(OH)D	RIA	80/80	7
11	Czernobilsky [34]	1988	Germany	1988	NA	25(OH)D_3_	CPBA	38/60	9
12	Bouillon [35]	1980	Belgium	1979	41	25(OH)D_3_	CPBA	23/81	7

### Findings from the meta-analysis

**Fig. 2:**
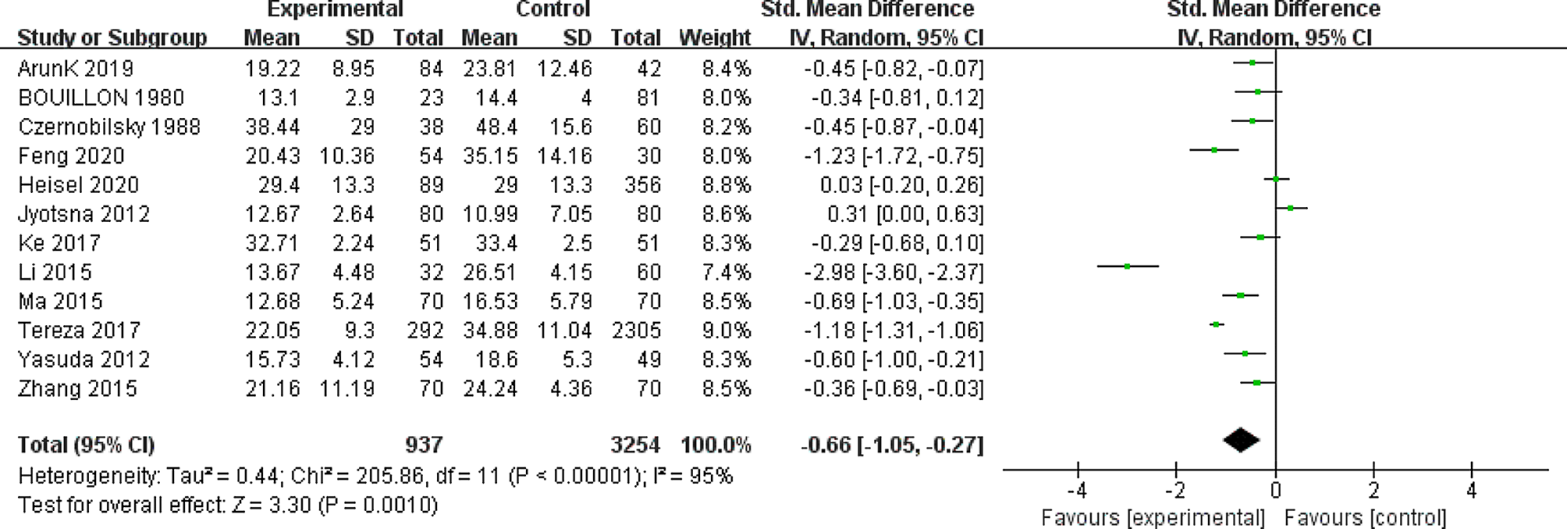
Forest plot of the relationship between vitamin D levels and Graves’ disease

The meta-analysis encompassed 12 studies involving 937 cases and 3254 controls, evaluating serum VD levels in patients with GD and healthy controls. These trials exhibited high heterogeneity in their results (chi-square = 205.86, *p* < 0.00001; I^2^ = 95%). Consequently, a random-effects model was applied for statistical analysis. The overall meta-analysis showed a pooled effect of SMD = − 0.66 (95% CI: −1.05, − 0.27; *p* = 0.001), supporting the association between low VD levels with GD, significantly different from the control group (Fig. 2). Sensitivity analysis revealed that excluding a single experiment, no impact was noted on the overall outcome.

### Subgroup analysis

**Fig. 3:**
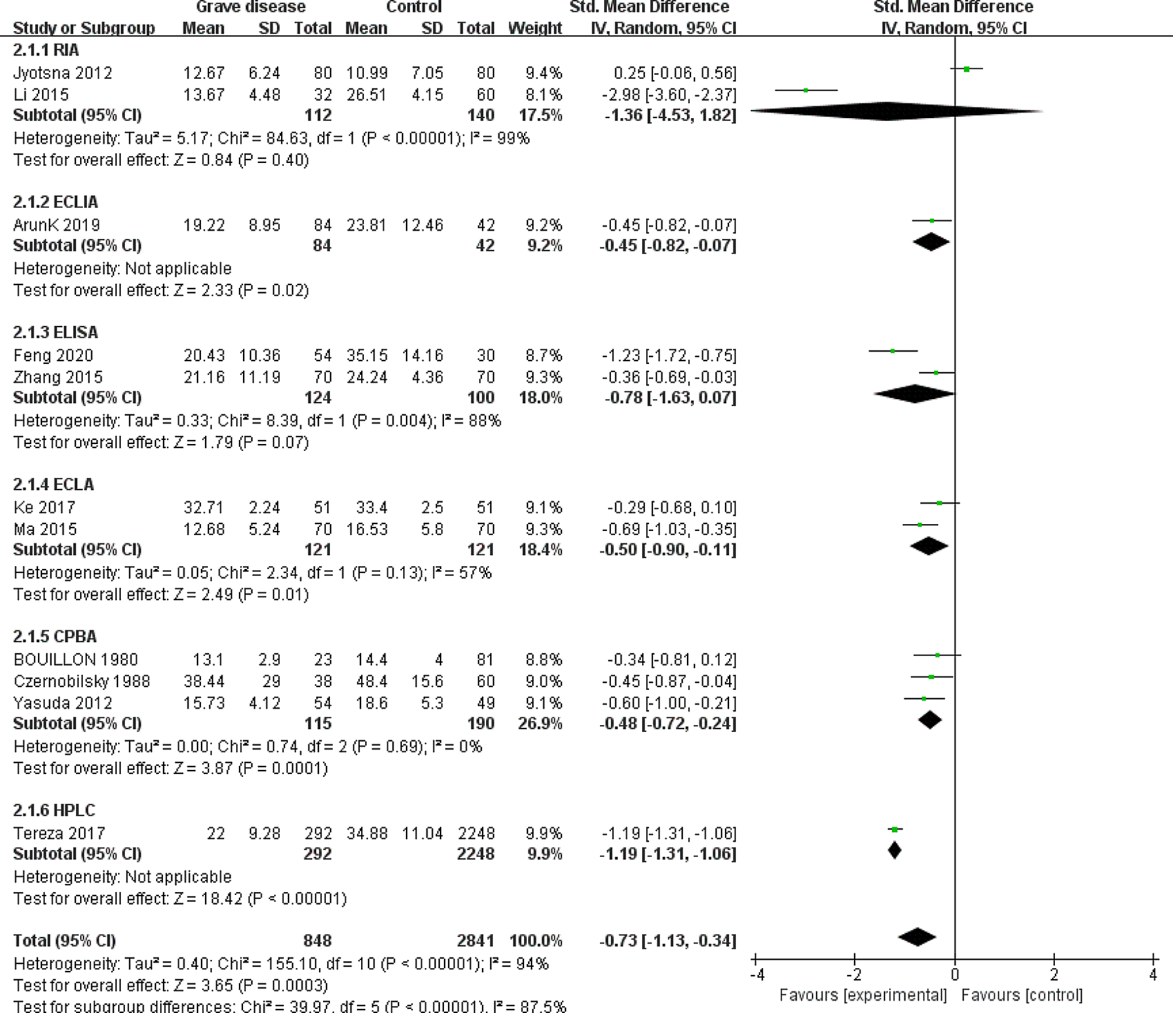
Forest plot of the subgroup of assay methods

**Fig. 4:**
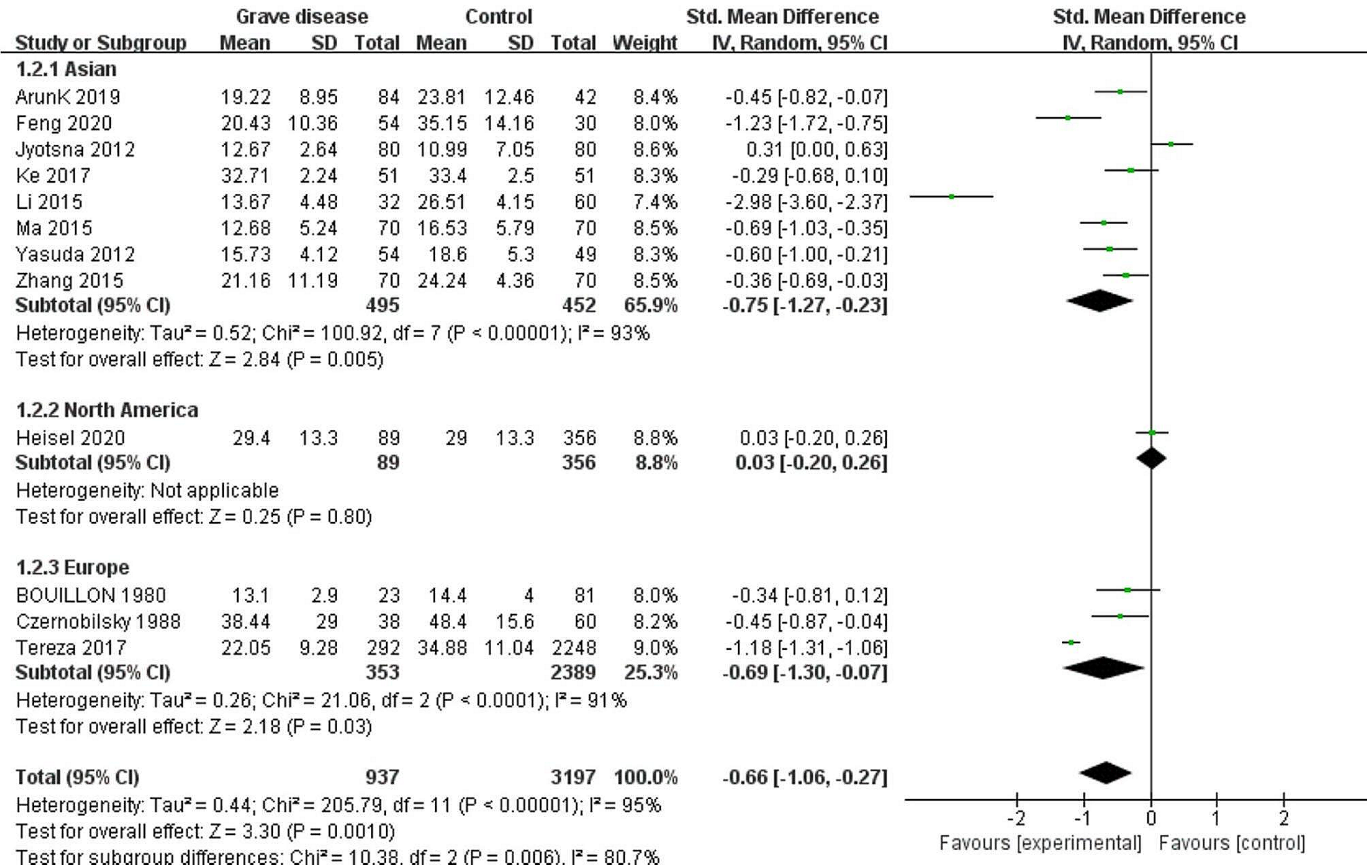
Forest plot of the subgroup of geographic location

**Fig. 5:**
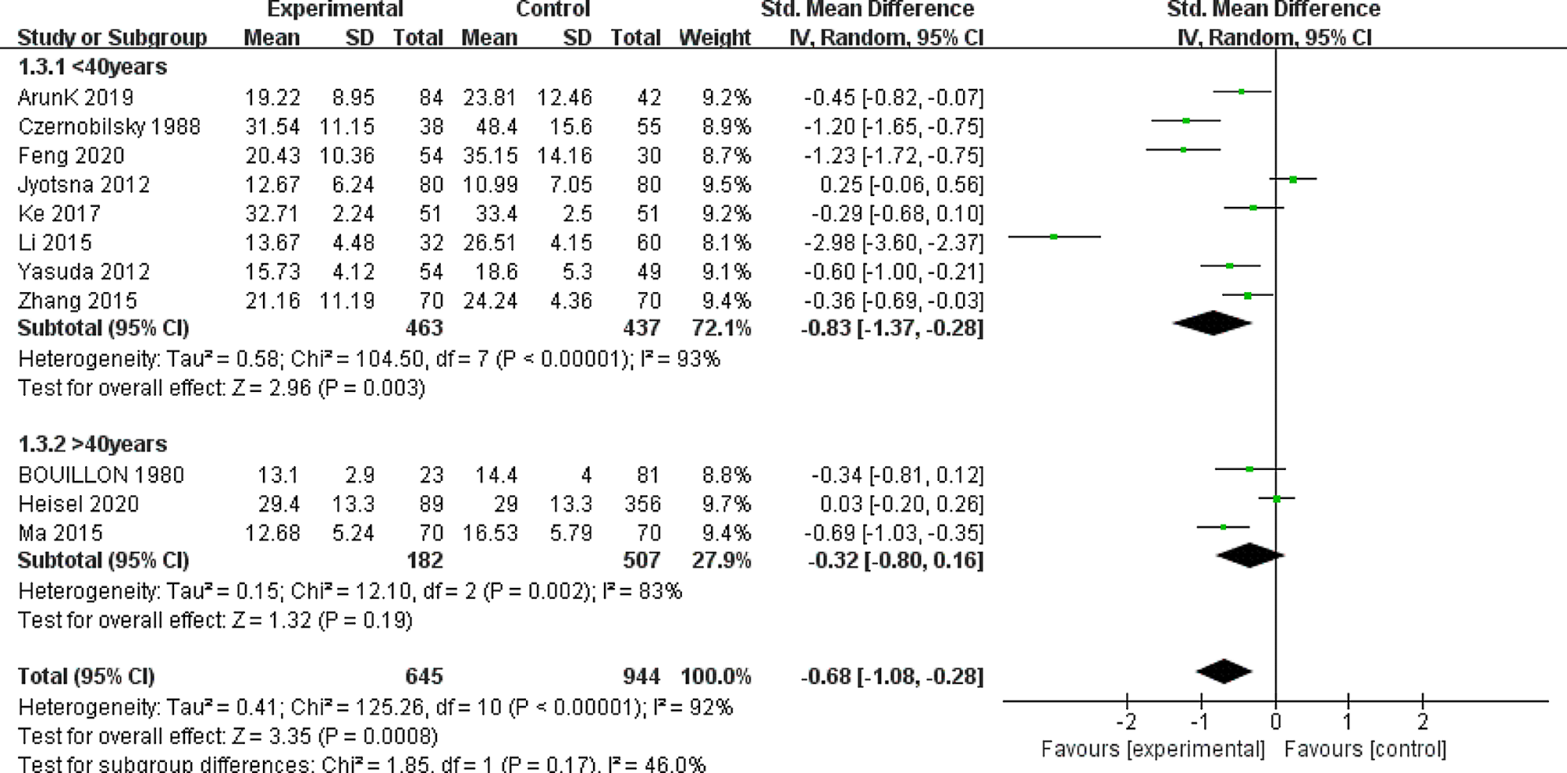
Forest plot of the subgroup of mean age

To identify sources of heterogeneity, we conducted subgroup analysis based on three factors: assay method (ELISA/ECLIA/HPLH/ECLA/CPBA/RIA; Fig. 3), geographic location (Asia/Europe/North America; Fig. 4), and mean age of the case group (≥ 40/<40 years; Fig. 5).

The subgroup analysis based on assay methods revealed that heterogeneity remained high (> 75%), even with a slight overall decrease (I^2^ = 94%). Among the six study groups, significant statistical differences were observed (*p* < 0.00001). Notably, heterogeneity in the CPBA group reduced to 0%, indicating lower heterogeneity than for the other assay methods. CPBA also showed a significant pooled effect (SMD = − 0.48; 95% CI: −0.72, − 0.24), favoring low VD levels in patients with GD. The pooled effect in the CPBA group was statistically significant (*p* = 0.0001). Heterogeneity in the ECLA group reduced to 57%, with an SMD of − 0.50 (95% CI: −0.90, − 0.11). RIA showed a higher heterogeneity (I^2^ = 99%) than the other assay methods, with an SMD of − 1.36 (95% CI: −4.53, 1.82), although the pooled effect was not statistically significant (*p* = 0.40). ELISA had an SMD of − 0.78 (95% CI: −1.63, 0.07) with high heterogeneity (I^2^ = 88%), and the pooled effect was not significant (*p* = 0.07). ECLIA and HPLC had significant pooled effects (SMD = − 0.45; 95% CI: −0.82, − 0.07; *p* = 0.02 for ECLIA and SMD = − 1.19; 95% CI: −1.31, − 1.06; *p* < 0.00001 for HPLC). In summary, assay method is a source of heterogeneity.

The subgroup analysis based on geographic location showed a pooled effect of SMD − 0.66 (95% CI: −1.06, − 0.27), with high heterogeneity (I^2^ = 95%). The subgroup of North America had a non-significant pooled effect (SMD = 0.03; 95% CI: −0.20, 0.26; *p* = 0.80), whereas Asia (SMD = 0.75; 95% CI: −1.27, − 0.23) and Europe (SMD = 0.69; 95% CI: −1.30, − 0.07) subgroups favored low VD levels in patients with GD, although the heterogeneity remained high (I^2^ = 93%, I^2^ = 91%, respectively). Thus, geographic location is a source of heterogeneity.

Subgroup analysis based on mean age showed a pooled effect of SMD − 0.68 (95% CI: −1.08, − 0.28), with high heterogeneity (I^2^ = 92%). The < 40 age group had a significant pooled effect (SMD = − 0.83; 95% CI: −1.37, − 0.28; *p* = 0.003), whereas the > 40 age group did not (SMD = − 0.32; 95% CI: −0.80, 0.16; *p* = 0.19). There were no statistically significant differences based on mean age (*p* = 0.17).

### Publication bias

**Fig. 6:**
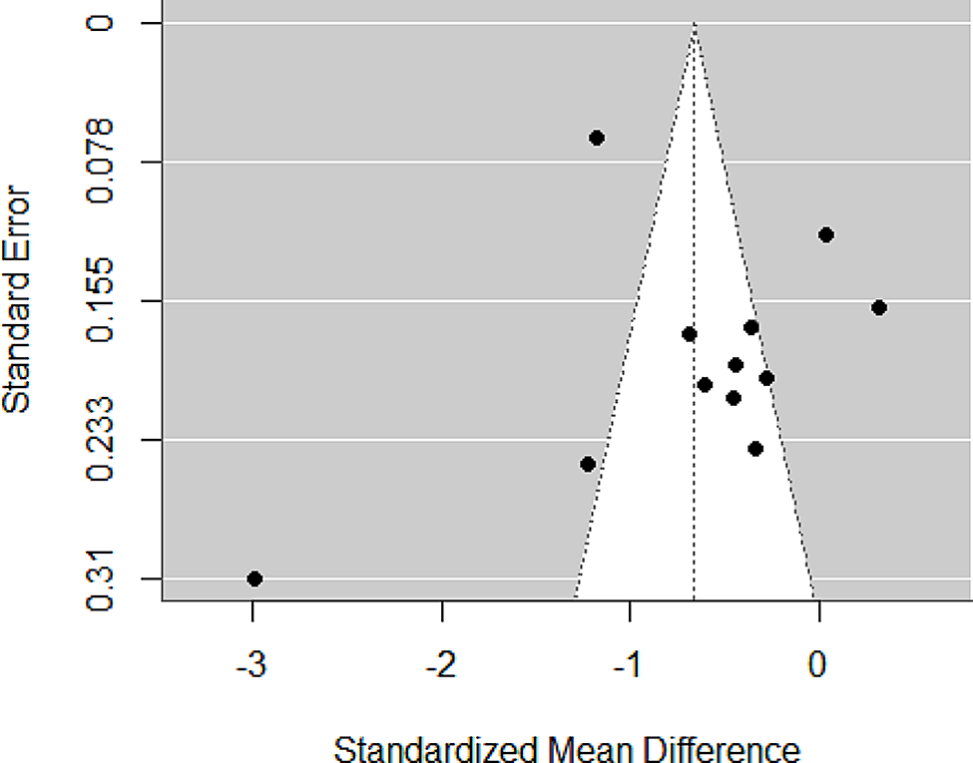
Funnel plot assessing the publication bias

The funnel plot (Fig. 6) and Egger’s test results (*p* = 0.0791) suggested no publication bias.

## Discussion

In our meta-analysis, we focused solely on investigating the relationship between quantitative data on serum VD levels and the onset of GD, without analyzing or discussing qualitative data on VD deficiency. This approach was chosen due to two main reasons: First, there is currently no unified criterion for assessing VD deficiency. For example, the American Endocrine Society considers serum 25(OH)D levels < 50 nmol/L as indicative of VD deficiency [36], whereas the Institute of Medicine suggests that serum 25(OH)D levels < 30 nmol/L indicate a risk of deficiency [37]. Second, some studies have reported a potential association between the onset of GD and low serum VD levels, but not with the prevalence of VD deficiency [19, 26]. Therefore, we did not focus on the relationship between VD deficiency and the onset of GD.

VD may influence the development of GD by regulating T and B lymphocytes. T cells have the ability to transform into regulatory T cells, T helper (Th) cells, and other specialized subtypes. Depending on the pattern of cytokine production, Th lymphocytes can be categorized into two subgroups: Th1 and Th2 [12]. Th1 supports cell-mediated immunity, whereas Th2 is involved in humoral immunity. Th1 lymphocytes secrete IFN-γ and IL-2, which are closely related to cell-mediated immune responses, whereas Th2 lymphocytes secrete IL-4 and IL-5, which are associated with humoral immunity [38]. The Th1 immune response prevails in the immune-pathogenesis of GD [7]. Tumor necrosis factor (TNF)-α, IFN-γ, and IL-2, which belong to pro-inflammatory Th1 cytokines, play pivotal roles in the pathogenesis of GD in the active state. In the initial stages of GD, Th1 cells are activated when resident follicular epithelial cells produce CXCL10, which intensifies inflammation [39]. Some studies have indicated that the Th1 immune response plays a critical role in the immune-pathogenesis of active phase of GD, whereas the inactive or later phases are associated with Th2 immune response [40, 41]. A previous study demonstrated that Th2-induced humoral immune response plays an important role in the pathogenesis of GD [42], as hyperthyroidism in GD. Studies have also demonstrated that hyperthyroidism in GD is caused by a Th2-mediated humoral response against the TSH receptor with stimulatory antibodies [42]. GD is directly related to B lymphocytes, as its specific antibody TRAb is synthesized by B cell clones in response to an autoimmune reaction. GD is most directly related to B lymphocytes, as its specific antibody TRAb is synthesized by B cell clones as a result of an autoimmune response.

Known as a liposoluble prohormone, VD comprises five distinct vitamins that get converted in the liver and kidneys into metabolically active forms [43, 44]. The most stable form of VD in serum is calcidiol [25(OH)D], which is used to assess VD status [44]. The active hormone form of VD is calcitriol [1,25(OH)2D], which is converted from 25(OH)D by most organs at the cellular level [45]. When 1,25(OH)2D interacts with its nuclear receptor (VD receptor [VDR]), ), it influences the expression of genes related to immunity, apoptosis, cell differentiation, and calcium metabolism [46]. Many immune cells not only express VDR but also can activate 25(OH)D into 1,25(OH)D [47].

Focusing on specific immune cells, we found that VD is closely related to Th1- and B cell immunity. VD can decrease the level of inflammatory Th1 cytokines [48] by inhibiting IFN-γ, IL-2, and IL-12, thereby negatively regulating Th1 cells [44]. Additionally, in the immune system, VD can regulate B cell function by reducing TRAb production via the inhibition of B cell proliferation and differentiation into plasma cells, suppressing the secretion of immunoglobulins (IgG and IgM), hindering the generation of memory B cells, and inducing apoptosis in B cells [13]. Thus, VD may attenuate GD by negatively regulating Th1 and B lymphocytes.

Applying the prior meta-analysis as a foundation, we further examined the association between serum VD levels and GD [21]. The scientific literature regarding the association between serum VD levels and GD was systematically evaluated and statistically summarized in this study. Although there is high heterogeneity in this study, a comprehensive analysis indicated that serum VD levels are lower in patients with GD than in healthy individuals.

The status of VD can be assessed using various VD assays, such as RIA, ECLIA, HPLC, CPBA, ELISA, and ECLA. Indeed, several studies have indicated high variability between different assays [49]. Due to the great variability in results reported by different methods, measuring 25(OH)D has proven to be a significant challenge [50]. In our subgroup analysis, the RIA subgroup showed high heterogeneity (I^2^ = 99%), whereas the CPBA subgroup showed low heterogeneity (I^2^ = 0%). These differing subgroup results may be related to the characteristics of different assays. RIA was first reported as detection method for VD in the 1980s, initially utilizing a ^3^H-labeled 25(OH)D tracer and subsequently developing into a ^125^I-labeled 25(OH)D assay with higher throughput and improved performance [51, 52]. As an immunoassay, the accuracy of RIA depends on the recognition capability of the antibodies used for VD [53]. Meanwhile, the accuracy of some RIAs could be affected by the types of VD (25(OH) D2 or 25(OH) D3) [54]. Thus, we considered the high heterogeneity of RIA to be associated with the antibody accuracy and types of VD. As the first automated method developed for 25(OH)D measurements, CPBA was published in 1971 [55]. The advantages of CPBA are low cost, small sample size capability, and co-specificity for different types of VD [53]. Thus, the co-specificity for different types of VD may indicate the low heterogeneity of CPBA. Therefore, the assay method is a source of heterogeneity.

VD can be obtained through exposure to sun’s ultraviolet (UV) light and diet [56]. However, it is not naturally present in many foods. The diet provides just under 5% of VD; therefore, UV exposure always been considered the major source of VD. Moreover, the entire requirement of VD can be satisfied via the exposure of our skin to UV radiation [57]. The level of UV radiation is significantly influenced by geographical factors, such as latitude and altitude. Increasing altitude leads to an increase in the intensity of UV radiation, whereas increasing latitude results in a decrease in the intensity [57, 58]. Geographic location significantly influences serum 25(OH)D levels [59], most likely due to differences in the intensity of UV radiation between different geographic areas. European countries included in this study have higher latitudes and lower average altitudes than Asian countries. Therefore, based on the geographical factors influencing UV exposure, serum VD levels in the Asian countries included in the study should be higher than those in the European countries. However, interestingly, the SMD of VD in Asia was lower than that of Europe. In addition, both continents showed high heterogeneity, which may be associated with differences in lifestyle habits and racial disparities. First, in terms of lifestyle habits, variations in sun exposure behaviors and lengths of time spent outside are the primary causes of individual variations in outdoor UV exposure. Moreover, personal outdoor UV exposure is an important influencing factor in VD research [57]. Urbanization often leads to insufficient outdoor activity, resulting in a reduction in the amount of personal outdoor sun exposure time [60]. In urbanized living, although we may receive sunlight exposure indoors and be exposed to UV radiation, this exposure does not facilitate VD synthesis but rather promotes VD degradation. Windows effectively block UV-B, which is essential for VD synthesis, exposing us to UV-A, which can break down VD [61, 62]. However, excessive exposure to UV-B radiation can lead to cancer; thus, exposure time should be managed flexibly [63]. However, excessive exposure to UV-B radiation can lead to cancer, so exposure time should be managed flexibly [63]. Furthermore, racial disparities are based on previous research that reported varying serum VD levels among different ethnicities in the same geographical location [64]. Skin color, characteristic of race, is determined by melanin produced by melanocytes in the epidermal layer of the skin. Melanin is an effective natural sunscreen that filters UV radiation [65]. Some studies have indicated that the threshold for VD production in the skin varies among different ethnicities, such as the threshold is higher in Asians than in Caucasians [66]. We believe that the geographic location is a source of heterogeneity, when dealing with individuals of the same ethnic group, whereas when dealing with individuals from different ethnic groups, whether geographical location is a source of heterogeneity needs to be discussed separately.

The results of the subgroup analysis for North America are interesting, with the pooled effect showing a negative outcome, not favoring low VD levels in patients with GD. We believe this outcome is associated with two factors. First, serum VD levels in health individuals in North America may be low. Previous research [67] indicated that VD deficiency is more prevalent in northern latitudes (Canada and the northern p.a.*r*t of the USA), which could contribute to these results. Further research is needed to clarify the findings of the North American subgroup, including expanding the sample size and conducting more accurate population studies. Second, the use of VD supplements is common in North American populations [56, 68]. . Thus, GD patients in North America may have the same VD levels as normal individuals as a result of supplement use. This result contrasts with the findings reported in other groups. Notably, nutritional supplementation policies vary among different countries, which can influence the VD levels in a region [56].

In our study, age was also considered as a possible cause of the high degree of heterogeneity. We found that VD levels did not significantly differ across subgroups of age, contrary to previous findings [23]. Although 25(OH)D levels typically decrease from birth to adolescence and stabilize during adulthood [69], the use of supplements was not considered. With increasing age, there is a growing trend of supplement usage, particularly in the > 40 age group, which may explain the subgroup analysis results in our study.

The efficacy of VD in regulating immune function has garnered increasing interest regarding its impact on GD.

In summary, our study conducted a systematic review and meta-analysis to assess the correlation between serum VD levels and GD. The results suggest that low serum VD levels are a risk factor for GD, indicating that VD supplementation could be a novel strategy for preventing and treating GD. Future clinical trials should delve deeper into this potential treatment approach. Considering the influence of geographic and ethnic differences on VD intake levels, future studies should involve multicenter, multiethnic clinical trials. Additionally, establishing unified evaluation standards for assessing VD deficiency across different regions is crucial.

### Limitations

Due to the high heterogeneity in our study, we conducted subgroup meta-analyses to explore the impact of geographic location, detection methods, and age on the association between serum VD levels and GD. The inclusion of only English-language studies journals indexed in the Science Citation Index led to a relatively limited number of studies in our analysis. Moreover, the lack of standardized criteria for evaluating VD deficiency and its weak association with the onset of GD were not explored in our study.

## Conclusion

Our meta-analysis further confirms that low VD levels may increase the risk of GD. Larger and more comprehensive clinical research is necessary to determine if VD insufficiency contributes to the onset of GD and whether VD supplementation is a viable treatment strategy for GD.

## Data Availability

All the data in this meta-analysis are from published studies and we take responsibilities for the data integration process and the accuracy of the statistical analyses process.

## Abbreviations

GD: Graves’ disease
TRAb: Thyrotropin Receptor Antibody
TSH: Thyroid Stimulating Hormone
RAI: Radioactive Iodine
ATDs: Antithyroid Drugs
VD: Vitamin D
IFN-γ: Interferon-γ
IL: Interleukin
AITD: Autoimmune Thyroid Disease
TSAb: Thyroid Stimulating Antibody
SMD: Standardized Mean Difference
CI: Confidence Interval
CPBA: Competitive Protein Binding Assays
ELISA: Enzyme-Linked Immunosorbent Assay
ECLIA: Electrochemiluminescence Immunoassay
RIA: Radioimmunoassay
ECLA: Euglobulin Clot Lysis Assay
HPLC: High-Pressure Liquid Chromatography
Th: T Helper
TNF: Tumor Necrosis Factor
Th2: Type 2 T Helper
VDR: VD Receptor
UV: Ultraviolet
UV-B: Ultraviolet Radiation B
UV-A: Ultraviolet Radiation A
